# Guidelines for Therapeutic Drug Monitoring of Vancomycin: A Systematic Review

**DOI:** 10.1371/journal.pone.0099044

**Published:** 2014-06-16

**Authors:** Zhi-Kang Ye, Can Li, Suo-Di Zhai

**Affiliations:** 1 Department of Pharmacy, Peking University Third Hospital, Beijing, China; 2 Department of Pharmacy Administration and Clinical Pharmacy, School of Pharmaceutical Sciences, Peking University Health Science Center, Beijing, China; University of Calgary, Canada

## Abstract

**Background and Objective:**

Despite the availability of clinical practice guidelines (CPGs) for therapeutic drug monitoring (TDM) of vancomycin, vancomycin serum concentrations still do not reach therapeutic concentrations in many patients. Thus, we sought to systematically review the quality and consistency of recommendations for an international cohort of CPGs regarding vancomycin TDM.

**Methods:**

PubMed, Embase, guidelines' websites and Google were searched for CPGs for vancomycin TDM. Two independent assessors rated the quality of each CPG using the Appraisal of Guidelines for Research & Evaluation II (AGREEII) instrument and data were independently extracted.

**Results:**

Twelve guidelines were evaluated and the overall quality of guidelines for vancomycin TDM was moderate. The highest score was recorded in the domain of clarity of presentation, and the lowest score was recorded in the domain of rigor of development and stakeholder involvement. The specific recommendations for vancomycin TDM were moderately consistent and guidelines varied in trough concentration monitoring, frequency of TDM, and serum concentration targets.

**Conclusion:**

The overall guideline quality for vancomycin TDM was not optimal and effort is needed to improve guideline quality, especially in the domain of rigor of development and stakeholder involvement.

## Introduction

Vancomycin is a first-line therapy for methicillin-resistant *Staphylococcus aureus* (MRSA) [1] and this drug is recommended for therapeutic drug monitoring (TDM) to minimize the risk of nephrotoxicity and to ensure successful therapeutic outcomes [2]. To improve the quality of vancomycin TDM, several organizations have developed clinical practice guidelines (CPGs) for appropriate vancomycin TDM. More patients have appropriate trough concentration measurement and sample timing when the guideline is followed [3]. However, many studies suggest that significant numbers of patients do not achieve therapeutic vancomycin serum concentrations [4]–[13].

CPGs are “statements that include recommendations intended to optimize patient care. They are informed by a systematic review of evidence and an assessment of the benefits and harms of alternative care option” [14]. Properly developed, high quality CPGs should offer better patient outcomes, reduce risk, and allow cost-effective clinical care [15], [16]. However, many CPGs offer poor quality, highly variable recommendations [17]–[21]. To our knowledge, a systematic evaluation of the quality and the consistency of vancomycin TDM guidelines have not been reported. Thus, the objective of this review was to systematically evaluate the quality and consistency of recommendations for an international cohort of CPGs regarding vancomycin TDM, and in an effort to help develop or update vancomycin TDM guidelines to achieve higher quality recommendations.

## Methods

### Identification of Guidelines

Guidelines for vancomycin TDM were identified (until June 25, 2013) in PubMed and Embase. Search terms included text words and Medical Subject Headings (MeSH) terms as follows: (“guideline” or “practice guideline” or “guidelines” or “practice guidelines” or “recommendation” or “consensus review” or “guideline” as TopicMeSH) and (“vancomycin” MeSH) and (“therapeutic drug monitoring” or “TDM” or “drug monitoring” or “therapeutic monitoring” or “serum concentration monitoring” or “therapeutic drug” or “drug monitoring” MeSH). Guideline websites and Google were searched to include more relevant CPGs: these included the National Guideline Clearinghouse (www.guideline.gov), Guidelines International Network (www.g-i-n.net/), National Institute for Health and Clinical Excellence (www.nice.org.uk), Scottish Intercollegiate Guidelines Network (www.sign.ac.uk) and China Guideline Clearinghouse (cgc.bjmu.edu.cn:820/). The search term was “vancomycin” and all results were reviewed. Google was searched using the words “vancomycin” and “guideline” and the first 100 items were reviewed. To ensure that all potentially relevant guidelines were retrieved, we conducted a search by country in Google and no language restriction was applied.

### Selection of Guidelines

CPGs for vancomycin TDM included those that both provided practical clinical recommendations and were endorsed by medical specialty associations, relevant professional societies or governmental agencies. Documents lacking such recommendations and secondary publications were excluded.

### Evaluation of Guidelines

Two assessors (Z.K.Y and C.L) used online training tools recommended by the AGREE collaboration before conducting appraisals. Two assessors independently scored each guidelines using AGREE II [22]. AGREE II consists of 23 items organized into six domains: “scope and purpose” (3 items), “stakeholder involvement” (3 items), “rigor of development” (8 items), “clarity of presentation” (3 items), “applicability” (4 items), and “editorial independence” (2 items). Each item is scored from 1 (strongly disagree) to 7 (strongly agree). We referred to methods of a previous study to resolve discrepancies between the two assessors: Briefly, if scores by both assessors differed by two points, they were averaged but if they differed by one point, the lower score was kept. Next, if scores between assessors varied by three points or more, a consensus was reached after a discussion. If consensus was not reached, a third person (S.D.Z) participated in the discussion and resolved the discrepancy [20]. The standard score of each domain was calculated as a percentage of the maximum possible score:
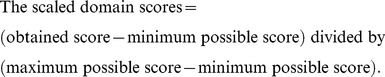
A score of 50% was chosen to establish the proportion of guidelines which scored greater than or equal to the level in six domains. The overall assessment of included CPGs was based on the overall quality of each guideline.

### Synthesis of results

The included CPGs were summarized according to specific recommendations, including indications for TDM, pharmacokinetics-pharmacodynamics, methods of TDM, target of serum concentrations and initial administration plan.

## Results

### Study selection

**Figure 1:**
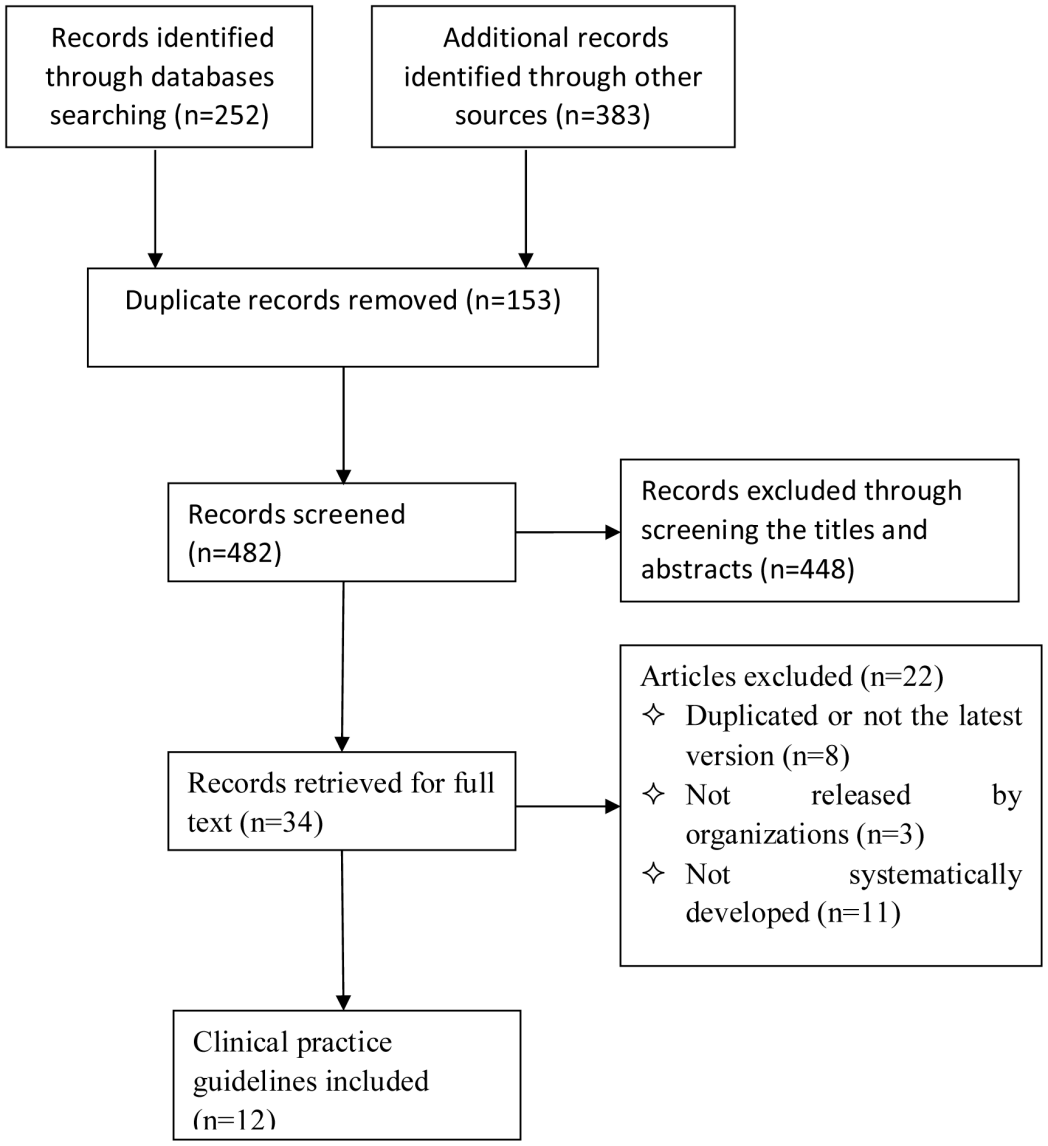
Flow chart for the systematic review.

Figure 1 shows the study selection process for inclusion in this review. A total of 635 records were retrieved and after application of the inclusion and exclusion criteria, 12 CPGs (AME [23], LOS [24], JAP [25], VAN [26], ALB [27], NHS [28], CAL [29], DEV [30], COR [31], BAT [32], SAP [33], WOR [34]) were included in the review. Table 1 depicts the demographic characteristics for included guidelines. Among the twelve CPGs, three (AME, JAP, NHS) were national CPGs [23], [25], [28], and the remaining CPGs were regional guidelines. The AME and JAP CPGs were found in medical literature databases [23], [25], and the others were found by Google searches. The AME and JAP CPGs rated the quality of evidence and graded the strength of recommendations using the classification schemata of the Canadian Medical Association.

**Table 1:** Characteristics of clinical practice guideline.

Title	Year of publication	Country/Region	Level of development	Organization behind the guideline	Number of authors	Number of references
Therapeutic monitoring of vancomycin in adult patients: A consensus review of the American Society of Health-System Pharmacists, the Infectious Diseases Society of America, and the Society of Infectious Diseases Pharmacists (AME) [23]	2009	America	National	ASHP/IDSA/SIDP	15	129
Vancomycin dosing and monitoring of serum vancomycin levels Infectious diseases section guidelines (LOS) [24]	2013	Los Angeles	Regional	VAGLAHS	NR	20
Practice guidelines for therapeutic drug monitoring of vancomycin: a consensus review of the Japanese Society of Chemotherapy and the Japanese Society of Therapeutic Drug Monitoring (JAP) [25]	2013	Japan	National	JSC/JSTDM	18	116
Vancomycin Therapeutic Drug Monitoring Vancouver Coastal Health & Providence Health Care Regional Guideline (VAN) [26]	2011	Canada, Vancouver	Regional	VCH/PHC	9	7
Vancomycin Monitoring and Dosing Guideline (ALB) [27]	2011	Canada, Edmonton	Regional	AHS	NR	11
Vancomycin Guideline for Adults (NHS) [28]	NR	United Kingdom	National	File NHS ADTC	NR	NR
Prescribing Guidelines for Intravenous Vancomycin in Adults (CAL) [29]	2009	United Kingdom, Calderdale and Huddersfield	Regional	CHNHS	NR	7
Guidelines on Intravenous (IV) Vancomycin Dosing in Adults (DEV) [30]	2010	United Kingdom, Devon and Exeter	Regional	RDENHS	NR	NR
Vancomycin prescription and therapeutic drug monitoring guideline (COR) [31]	2010	United Kingdom, Cornwall	Regional	RCHNHS	7	3
Guidelines for the Dosing and Monitoring of Gentamicin, Vancomycin and Teicoplanin (BAT) [32]	2009	United Kingdom, Bath	Regional	RUHBNHS	NR	6
Intravenous Vancomycin Use in Adults Intermittent (Pulsed) Infusion (SAP) [33]	2013	United Kingdom, Scottish	Regional	SAPG	NR	NR
Guidelines for Vancomycin Dosing and Monitoring in Adult Patients (WOR) [34]	2008	United Kingdom, Worcestershire	Regional	WAHNHS	10	5

AME: American; ASHP: American Society of Health-System Pharmacists; IDSA: Infectious Diseases Society of America; SIDP: Society of Infectious Diseases Pharmacists; LOS: Los Angeles; VAGLAHS: VA Greater Los Angeles Healthcare System; JAP: Japanese; JSC: Japanese Society of Chemotherapy; JSTDM: Japanese Society of Therapeutic Drug Monitoring. VAN: Vancouver; VCH: Vancouver Costal Health; PHC: Providence Health Care; AHS: ALB: Alberta; Alberta Health Services; NHS: National Health Services; File NHS ADTC: File National Health Services Board Area Drugs and Therapeutics Committee; CAL: Calderdale; CHNHS: Calderdale and Huddersfield NHS; DEV: Devon; RDENHS: Royal Devon and Exeter NHS; COR: Cornwall; RCHNHS: Royal Cornwall Hospitals NHS; BAT: Bath; RUHBNHS: Royal United Hospitals Bath NHS; SAP: Scottish Antimicrobial Prescribing; SAPG: Scottish Antimicrobial Prescribing Group; WOR: Worcestershire; WAHNHS: Worcestershire Acute Hosptials NHS; NR: not reported.

### Scope and Purpose

Table 2 shows the standardized scores of each domain and overall recommendation. The mean score for the domain of scope and purpose was 63% (range 28–100%). Nine guidelines scored greater than or equal to 50% [23], [25]–[27], [29]–[31], [33], [34], two of them scored greater than or equal to 94% [23], [25]. Most guidelines clearly specifically described their scope, related clinical questions and target populations.

**Table 2:** AGREE II domain-standardized scores for CPGs on vancomycin TDM.

Guideline	Scope and Purpose (%)	Stakeholder Involvement (%)	Rigor of development (%)	Clarity and presentation (%)	Applicability (%)	Editorial independence (%)	Overall assessment
AME	100	50	71	100	54	67	Recommend
LOS	39	6	4	78	38	25	Not recommend
JAP	94	50	73	100	58	67	Recommend
VAN	89	33	13	78	54	42	Recommend with modification
ALB	50	17	13	94	54	42	Recommend with modification
NHS	28	11	4	61	42	33	Not recommend
CAL	50	11	4	78	42	42	Not recommend
DEV	50	22	8	56	46	42	Not recommend
COR	83	50	13	72	46	50	Recommend with modification
BAT	33	17	8	73	46	42	Not recommend
WOR	78	44	19	67	42	50	Recommend with modification
SAP	56	17	6	72	46	42	Not recommend
Mean (Range)	63 (28–100)	27 (6–50)	20 (4–73)	77 (56–100)	47 (38–58)	45(25–67)	

### Stakeholder Involvement

The mean score for the domain of stakeholder involvement was 27% (range 6–50%). Only three guidelines scored 50% [23], [25], [31]. No guidelines appeared to include or consider the views or preferences of the target population. Also, members of the guideline development group were not well identified for many guidelines.

### Rigor of development

The mean score for the domain of rigor of development was 20% (4–73%). Two guidelines scored above 70% [23], [25], the remaining guidelines scored below 20%. Only the AME CPG clearly described the systematic methods for searching evidence [23] and the JAP CPG clearly described the procedure of updating the guideline [25]. No guideline reported their recommendations on an underlying systematic review.

### Clarity of presentation

The mean score for the domain of clarity of presentation was 77%. All CPGs scored above 50%. Three CPGs scored greater than 90% [23], [25], [27]. Most guidelines presented specific, easily identified recommendations for the management of vancomycin TDM.

### Applicability

The mean score for the domain of applicability was 47% (range 38–54%). Only four CPGs scored greater than 50% [23], [25]–[27]. No guideline considered the cost of vancomycin TDM, and little information was offered to describe TDM barriers or facilitators.

### Editorial independence

The mean score for the editorial independence was 45% (25–67%). Four CPGs scored greater than or equal to 50% [23], [25], [31], [34]. Only the AME and JAP CPGs reported the information about competing interests of guideline development group members [23], [25].

### Clinical practice guideline recommendations

#### Indication of TDM

In Table 3, TDM indication reporting is described for the CPGs. Four CPGs (JAP, AME, ALB and VAN) recommended that TDM should be performed in patients receiving aggressive dosing, patients with high risk of nephrotoxicity, unstable renal function, and in those receiving prolonged therapy (more than three or five days). Three CPGs (JAP, VAN and ALB) specifically recommended that TDM should be performed in patients undergoing hemodialysis, those who were obese or had low body weight, those with special conditions that cause fluctuating volumes of distribution, and in pregnant and pediatric patients. The ALB CPG recommended vancomycin TDM should be performed in patients with anticipated therapy of more than two weeks, and the LOS CPG recommended that vancomycin TDM should be performed in patients receiving more than 48 h of vancomycin therapy (Table 3).

**Table 3:** Recommendations from CPGs.

Item	AME	LOS	JAP	VAN	ALB	NHS	CAL	DEV	COR	BAT	SAP	WOR
Indication of TDM	√	√	√	√	√	NR	NR	NR	NR	NR	NR	NR
PK–PD parameter	√	NR	√	√	NR	NR	NR	NR	NR	NR	NR	NR
Method of TDM												
Peak or trough concentration	trough	trough	trough	Pre–levels and post–levels	trough	trough	Pre–dose levels	Pre–dose levels	Peak and trough	Pre–dose levels	trough	trough
Time for trough sample	Within 30 min	NR	Within 30 min	NR	Within 30 min	NR	NR	Within 60 min	NR	NR	NR	NR
Time to first level (patients with normal renal function)	Before 4^th^ dose	Before 5^th^ dose	Before 4^th^ or 5^th^ dose	not earlier than 3^rd^ dose and within 48 h	After at least two dose	before 2^nd^ maintenance dose	Before 3^rd^, 4^th^, or 5^th^ dose	NA	before 3^rd^ or 4^th^ dose	Before 3^rd^ or 4^th^ dose	within 48 h of starting therapy	Before 3^rd^, 4^th^ dose
Frequency of TDM (patients with normal renal function)	weekly	Depend on clinical condition	weekly	weekly	weekly	Twice weekly	Twice weekly	weekly	After 4 days	Twice weekly	Every 2–3 days	Every 3–4 days
Target of trough concentration (µg/mL)	10–20	10–20	10–20	Lower than 20	5–20	10–20	10–20	10–20	10–15	5–15	10–20	5–15
Target trough concentration in complicated infections	15–20	15–20	15–20	15–20	10–20	15–20	15–20	NR	NR	10–15	15–20	Higher levels^a^
Loading dose	25–30 mg/kg	25–30 mg/kg	25–30 mg/kg	(ma×2,500 mg/dose)	NR	Loading dose	NR	Loading dose	NR	NR	Loading dose	NR

NR: not reported.

a Higher levels may be required in specific situations as directed by the microbiologist.

#### Pharmacokinetic and pharmacodynamics monitoring (PK-PD) parameters

Three CPGs (JAP, AME and VAN) recommended that an area under the curve (AUC)/minimum inhibitory concentration (MIC) ratio of more than 400 was associated with clinical efficacy of vancomycin therapy. Trough concentrations were the best surrogates for AUC. Other CPGs did not consider a monitoring parameter associated with clinical efficacy (Table 3).

#### Peak or trough concentrations

Ten CPGs (JAP, AME, LOS, ALB, NHS, CAL, DEV, BAT, SAP and WOR) recommended monitoring trough concentrations or pre-dose levels rather than peak serum concentrations. The VAN CPG recommended monitoring pre- and post-dose concentrations to obtain precise pharmacokinetics for some special patients. The COR CPG recommended monitoring peak and trough serum concentrations (Table 3).

#### Time to first sample

Most CPGs recommended obtaining the first trough sample at steady state (before the 3^rd^, 4^th^, or 5^th^ dose in patients with normal renal function). The SAP CPG recommended monitoring troughs within the 48 h of starting therapy. The DEV CPG did not report a time for obtaining the first trough (Table 3).

#### Frequency of TDM

Five CPGs (AME, JAP, ALB, VAN and DEV) recommended weekly monitoring after initial TDM in patients with normal renal function, and more frequent follow-up trough concentration monitoring was required in patients with hemodynamic instability, high-dose vancomycin administration, unstable renal function, and those at high risk for nephrotoxicity. The LOS CPG recommended more frequent monitoring in patients with complicated infections (goal trough was 15–20 µg/mL) or those with longer courses of therapy. Other CPGs recommended additional drug concentration measurements 4 days or less for patients with normal renal function, and recommended more frequent monitoring for patients with unstable renal function, hemodynamic instability, or in patients who experienced changes in renal function (Table 3).

#### Sample time

Three CPGs (JAP, AME and ALB) recommended a trough sample should be obtained within 30 min prior to next dose. The DEV CPG recommended a trough measurement within 60 min prior to the next dose. Other CPGs did not recommend trough sample timing (Table 3).

The VAN CPG defined a 3 or 24 h post-dose serum concentration as a “post levels”. The COR CPG recommended measuring peak concentrations 1 h after the end of infusion. In addition, the JAP CPG did not recommend routine monitoring peak concentrations, but if peak concentrations are needed in some special circumstances, peak concentrations should be measured 1–2 h after the end of infusion.

#### Target of serum concentrations in TDM

Only three CPGs (ALB, BAT and WOR) recommended that vancomycin trough concentrations should be more than 5 µg/mL, and most CPGs recommended that vancomycin trough concentrations should be maintained above 10 µg/mL to avoid development of drug resistance. Most CPGs recommended higher trough concentrations in patients with bacterial infections, infective endocarditis, osteomyelitis, meningitis and hospital-acquired pneumonia, but no CPG recommend trough concentrations greater than 20 µg/mL. The VAN CPG recommended 15–20 µg/mL for patients with complicated infections and suggested less than 10 µg/mL for patients with urinary tract infections or skin and soft tissue infections not due to MRSA. The ALB CPG recommended trough concentrations at 5–20 µg/mL. If therapy was combined with aminoglycosides, recommended trough concentrations were lower than those for patients without aminoglycoside combination therapy. The COR CPG recommended trough concentrations of 10–15 µg/mL, and peak concentrations of 18–26 µg/mL. The VAN CPG recommended 3 h post vancomycin concentrations of 20–40 µg/ml (Table 3).

#### Initial administration plan

All guidelines recommended calculating the vancomycin dose according to renal function. Three CPGs (JAP, LOS and AME) recommended giving a loading dose of 25–30 mg/kg to facilitate rapid attainment of target trough concentrations for serious or complicated infections. Four CPGs (VAN, NHS, DEV and SAP) recommended prescribing a loading dose according to the patients' actual body weight. Five CPGs (ALB, CAL, COR, BAT and WOR) did not recommend a loading dose (Table 3).

### Overall assessment

Two CPGs (AME, JAP) were recommended [23], [25], and four CPGs (VAN, ALB, COR and WOR) were recommended with modification [26], [27], [31], [34]. Six CPGs (LOS, NHS, CAL, DEV, BAT and SAP) were not recommended. The two CPGs that were recommended have a higher score in domain of rigor of development and a standard search strategy, and they classified the quality of evidence and graded the strength of recommendations. The six CPGs that were not recommended scored below 10% in the domain of rigor of development and the other domains' scores was not high.

## Discussion

To our knowledge, this is the first study to evaluate the quality and consistency of vancomycin TDM guidelines; although, CPG quality has been investigated in a variety of clinical areas [17]–[21]. We made three important findings: first, the overall guideline quality was moderate, and more efforts are needed to improve these guidelines, especially with respect to the domain of rigor of development and stakeholder involvement. Second, vancomycin TDM guideline recommendations were moderately consistent. Third, regional guidelines were of lower quality than national guidelines. In the United Kingdom and Canada, national guidelines may be of sufficient quality to replace regional guidelines of those areas.

Guidelines consistently scored well with respect to clarity and presentation, suggesting that this domain may be easier to achieve or may be more highly emphasized by guideline developers. The lowest score was recorded in rigor of development, perhaps due to the fact that most guidelines did not report the systematic methods for evidence searching, and many had poorly described information about selection criteria, evidential strengths and limitations, and procedures for updating guidelines. The AME and JAP CPG had the highest scores in this domain and all rated the quality of evidence and graded the recommendation strength, indicating that using a formal system might improve scores for developmental rigor. Developmental rigor is closely related to guideline quality and guideline developers should pay more attention to this domain. The mean score for stakeholder involvement was 27%. No guidelines have considered the views and preferences of patients or of the public, but patient involvement in decision making about care management may improve physician and patient guideline adherence and improve clinical outcomes [35].

The mean score for applicability was 47%, and only four guidelines scored greater than 50% in this area. No guideline considered the cost of vancomycin TDM and no guideline provided enough evidence to support the necessity of vancomycin TDM. Guideline developers did not address potential barriers of guideline implementation and this may have contributed to many hospitals not monitoring vancomycin serum concentrations and many patients not achieving target therapeutic concentrations. The mean score for the scope and purpose was 63%, and most guidelines described their scope, related clinical questions and target populations well. The mean score for editorial independence was 45%. No guidelines described funding sources, although they were developed by medical societies. Most guidelines did not offer data regarding competing interests among guideline development group members. Guideline developers should emphasize these points in future studies.

Specific recommendations of vancomycin TDM guidelines were moderately consistent and varied with respect to trough concentration monitoring, TDM frequency and target serum concentrations across guidelines, which was possibly attributed to unique references for each guideline and only two guidelines (AME, JAP) describing their systematic search strategy. Also, few prospective or randomized trials for vancomycin TDM were available and most of the published literature regarding vancomycin monitoring are observational studies.

The AME and JAP CPG rated the quality of evidence and graded recommendations using the same classification schemata recommended by the Canadian Medical Association. However, evidence and strength of recommendations were inconsistent, and this may be attributed to the search strategy, criteria for selecting evidence, methods for formulating recommendations, and experts' consensus [36]. The Grading of Recommendations Assessment, Development and Evaluation (GRADE) approach for rating the quality of evidence and grading the strength of recommendations is increasingly being adopted by organizations because this rating system is explicit, comprehensive, transparent and pragmatic [37].We advise guideline developers to adopt GRADE for this reason.

Our search identified all potentially relevant studies but limitations of our approach included the fact that included CPGs were written in English or Chinese. So other CPGs written in other languages were likely missed, even though no restriction on language was applied. Second, AGREE II did not provide criteria about the overall assessment to guide assessors in determining scores, so two assessors may fail to properly weigh domain scores.

In conclusion, the overall quality of vancomycin TDM guidelines was moderate and warrant improvement. Specifically, rigor of development and stakeholder involvement would benefit from increased scrutiny. Guideline recommendations were moderately consistent, especially with respect to regional guidelines. Local adaptation of existing high-quality CPGs to national use is worth considering and a national, high quality guideline to replace various regional guidelines would avoid duplicate efforts. The developers of guidelines should adhere more closely to the AGREE instrument when developing or updating vancomycin TDM guidelines.

## Supporting Information

Checklist S1 **PRISMA 2009 Checklist.doc.**
(DOC)
